# Epidemiological aspects of rheumatoid arthritis patients affected by oral bisphosphonate-related osteonecrosis of the jaws

**DOI:** 10.1186/1746-160X-8-5

**Published:** 2012-03-01

**Authors:** Nicolau Conte-Neto, Alliny Souza Bastos, Rosemary Adriana Chierici Marcantonio, Elcio Marcantonio Junior

**Affiliations:** 1 UNESP- Univ. Estadual Paulista, School of Dentistry, Department of Diagnosis and Surgery, Division of Periodontology, Rua Humaitá, 1680, 14801-903 Araraquara, SP, Brazil

**Keywords:** Rheumatoid arthritis, Bisphosphonate, Jaws, Osteonecrosis

## Abstract

This literature review aims to evaluate the epidemiologic profile of patients with rheumatoid arthritis (RA) that developed a bisphosphonate-related osteonecrosis that affect the jaws (BRONJ), including demographic aspects, as well as clinical and therapeutic issues. A search of PUBMED/MEDLINE, Scopus, and Cochrane databases from January 2003 to September 2011 was conducted with the objective of identifying publications that contained case reports regarding oral BRONJ in RA patients. Patients with RA who develop oral BRONJ are usually women above 60 years taking steroids and long-term alendronate. Most of them have osteoporosis, and lesions, triggered by dental procedures, are usually detected at stage II in the mandible. Although there is no accepted treatment protocol, these patients seem to have better outcomes with conservative approaches that include antibiotic therapy, chlorhexidine, and drug discontinuation.

## Background

Bisphosphonates (BPs) are stable synthetic analogs of inorganic pyrophosphate that suppress osteoclast-mediated bone resorption [1]. In this way, they have been widely used to stabilize bone loss in patients with rheumatoid arthritis (RA), especially those who develop osteoporosis, which is a common feature in this rheumatic disease [2]. However, since 2003, great concern has been generated regarding the side-effects of these drugs through increasing reports, worldwide, of a bisphosphonate-related osteonecrosis that affect the jaws (BRONJ).

There are many hypotheses regarding BRONJ pathogenesis, but none of them is completely accepted. Although there have been reports with no obvious co-morbidity factors [3,4], it is reasonable to believe that co-factors may play a relevant role in the development of these lesions, especially in patients taking oral BPs. Among these co-factors, RA has been included as a relevant risk factor for BRONJ; however, until now the relationship between these diseases has not been fully explained.

Due to the greater number of patients taking oral BPs for the treatment of RA and osteoporosis, it is very important for risks to be assessed. Thus, the purpose of this extended literature review is to evaluate relevant issues of patients with RA who developed oral BRONJ, including demographic, clinical, and treatment aspects, with the goal of establishing comparative associations with patients without RA who developed oral BRONJ.

## Methods

We performed a computerized search to identify all papers published in English from January 2003 to September 2011 in PUBMED/MEDLINE, Scopus, and Cochrane databases. Case reports, case series, and retrospective studies were included, while short communications and letters to the Editor were excluded. The studies were approved by the Ethics in Human Research Committee and were in compliance with the ethical principles of the Helsinki Declaration. Literature reviews and systematic reviews also were considered with the objective of identifying cases already reported. The key MeSH (Medical Subject Headings) terms used were: diphosphonates, bisphosphonate, jaw osteonecrosis, rheumatoid arthritis, and rheumatologic.

The primary objective was to identify all articles containing clinical reports with the following inclusion criteria:

• Diagnosis of RA and oral BRONJ

• Patients with no prior history of radiation to the craniofacial region

• Non-neoplasic patients

• At least five areas of the specific demographics were collected, including gender, age, bisphosphonate type and length of exposure, concomitant medications, trigger event, clinical features, staging, lesion site, imaging and histological features, as well as management and outcomes. The stage of the lesions was retrieved directly from the papers or it was defined based on the available clinical data and according to the classification system by Ruggiero et al. [5].

• Papers published in English

All titles and abstracts from the results of the literature search were reviewed by the authors for potential inclusion in our study. We also searched the related links of all relevant papers. Furthermore, all relevant papers were checked to avoid multiple inclusions of the same patients in this study.

## Results

The search strategy yielded 1606 titles/abstracts from the databases analyzed. After title, abstract screening, and/or paper analyses, 331 potentially relevant studies were identified and screened for retrieval. Of these, 312 studies were excluded due to non-compliance with the inclusion criteria, one paper was excluded due to duplicity of one patient and, finally, 17 were included in the present review, consisting of 27 patients and 29 BRONJ lesions (Tables 1, 2, 3, and 4).

**Table 1:** Patients and Bisphosphonate Therapy Characteristics

Authors/Study	Gender	Age	Comorbidities	Medications (Years)
Marunick et al. [4] CS *Patient 1*	F	59	OST	STE + METX + AL (3)

Yarom et al. [6] RS				
*Patient 2*	F	73	HTN	STE + METX + AL (7), infliximab
*Patient 3*	F	76	HTN + Arrhythmia + hypercholesterolemia	AL (1.5)

Malden & Pai [7] CS *Patient 4*	F	56	NO	STE + AL (1), leflunomide

Khamaisi et al. [8] RS *Patient 5*	F	73	DM	AL (> 0,5)

Marx et al. [9] CS				
*Patient6*	F	NA	OST	STE + METX + AL (3.1)
*Patient 7*	F	NA	OST	STE + METX + AL (6.3)
*Patient 8*	F	NA	OST	STE + METX + AL (3.3)

Hamada [10] CR				
*Patient 9*	F	68	OST	STE + RISE (4)

Barrow [11] CR				
*Patient 10*	F	70	OST	STE + AL (< 3)

Junquera et al. [12] CS				
*Patient 12a/12b*	M	73	NO	STE + METX + AL (3.8)

Mehanna et al. [13] CR				
*Patient 13*	F	55	NO	STE + Leflunomide + oral BPs (1)

Favia et al. [14] CS				
*Patient 14a/14b*	F	67	OST	STE + IBAN (1)

Kwon et al. [15] CR				
*Patient 15*	F	71	OST + DM	STE + AL (3); OH

Sedghizadeh et al. [16] RS				
*Patient 16*	F	63	OST + HTN	STE + chemotherapy + AL (3)

Lo et al. [17] RS				
*Patient 17*	NA	NA	NO	oral BPs (4.8)
*Patient 18*	NA	NA	Hip Osteonecrosis	STE + oral BPs (2.6)

Shin et al. [18] CR				
*Patient 19*	F	67	OST + HTN	AL (1) + HTN Drug

Park et al. [19] CS				
*Patient 20*	F	68	OST	STE + AL (5)
*Patient 21*	F	69	OST + PLE + TB	STE + AL (10)
*Patient 22*	F	70	OST + HTN	STE + AL (6)

Manfredi et al. [20] CS				
*Patient 23*	F	68	OST + DM + HTN	AL (4.25)
*Patient 24*	F	65	OST + HTN	AL (5)
*Patient 25*	F	79	OST	AL (4)
*Patient 26*	F	57	OST + DM + HTN	AL (> 3)

Conte-Neto et al. [21] CR				
*Patient 27*	F	58	NO	AL (5)
*Patient 28*	F	68	OST	STE + METX + AL (7)

*CS *Case series, *RS *Retrospective study, *CR *Case report, *NA *Not available

*OST *Osteoporosis, *HTN *Hypertension, *DM*, Diabetes Mellitus, *GU *gastric ulcer, *PLE *Pleuritis, *TB *Tuberculosis, *STE *Steroids, *MTX *Methotrexate; *AL *Alendronate, *RISE *Risedronate, *IBAN *Ibandronate, *OH *oral hypoglycemiant

**Table 2:** Clinical features of BRONJ lesions in patients with RA

Authors	Trigger event	Clinical features	Stage	Site
Marunick et al [4]				
*Patient 1*	Extraction	BE + pain + seqüestration	II	Md posterior lingual

Yarom et al [6]				
*Patient 2*	Implants	BE + pain + edema + PD + fistula + pathological fracture	III	Md posterior
*Patient 3*	Extraction	BE + NHS + pain + edema + PD + fistula	III	Md posterior

Malden & Pai [7]				
*Patient 4*	Extraction	BE + pain + NHS + edema	III	Mx posterior

Khamaisi et al [8]				
*Patient 5*	Dental surgery	NA	II	Md

Marx et al [9]				
*Patient 6*	Spontaneous	BE	I	Md lingual
*Patient 7*	Palatal CT graft	BE + erythema	II	Mx posterior palate
*Patient 8*	Spontaneous	BE	II	Md posterior lingual

Hamada [10]				
*Patient 9*	Spontaneous	Pain + edema + sequestration	II	Md

Barrow [11]				
*Patient 10*	Denture trauma	BE + pain + PD + erythema	II	Mx palatine

Junquera et al [12]				
*Patient 12a*	Extraction	BE + NHS + pain + hypoesthesia + erythema	II	Md anterior
*Patient 12b*	Spontaneous	BE + edema + trismus + pain + swelling	II	Md posterior lingual

Mehanna et al. [13]				
*Patient 13*	Spontaneous	BE + PD + trismus + abscess + pain	II	Md posterior

Favia et al. [14]				
*Patient 14a*	Extraction	BE + pain + PD	III	Mx posterior
*Patient 14b*	Extraction	BE + pain + PD	III	Md posterior

Kwon et al. [15]				
*Patient 15*	Spontaneous	BE + PD + edema	II	Mx and Md posterior

Sedghizadeh et al [16]				
*Patient 16*	Denture trauma	BE + pain + infection	II/III	Mx

Lo et al [17]				
*Patient 17*	Extraction	BE + PD edema	II	Md posterior lingual
*Patient 18*	Extraction	BE + pain + erythema	II	Mx posterior

Shin et al. [18]				
*Patient 19*	Implants	BE + pain + erythema + edema + increased probing depth	II	Mx posterior

Park et al [19]				
*Patient 20*	Implants	Pain + gingival bleeding + PI Pain	II	Md posterior
*Patient 21*	Extraction	+ PD + periodontitis	II	Mx posterior
*Patient 22*	Spontaneous	Pain + itching sensation + edema	III	Md posterior and anterior

Manfredi et al. [20]				
*Patient 23*	Spontaneous	BE + pain + infection	III	Md
*Patient 24*	Spontaneous	BE + pain + infection	II	Md
*Patient 25*	Spontaneous	BE + pain + infection	II	Md
*Patient 26*	Extraction	BE + pain + infection	II	Md

Conte-Neto et al. [21]				
*Patient 27*	Spontaneous	Pain + infection + increased probing depth + erythema	II	Md posterior
*Patient 28*	Spontaneous	BE + pain + infection + edema + erythema + increased probing depth + PD	II	Md posterior

*BE *Bone exposure, *PD *purulent discharge, *NHS *non-healing socket, *CT *connective tissue, *PI *periimplantitis, *Md *mandible, *Mx *maxillae

**Table 3:** Radiographic and Histologic Features of BRONJ Lesions in Patients with RA

Authors	Radiographic features	Histologic features
Marunick et al. [4]		
*Patient 1*	Osteolysis + sequestration	Dense nonvital bone + subacutely inflamed granulation tissue + bacterial colonies

Yarom et al. [6]		
*Patient 2*	ID	CLP inflammatory infiltrate + increased vascularity + necrotic bone + bacterial colonies (S. milleri)
*Patient 3*	ID	CLP inflammatory infiltrate + increased vascularity + necrotic bone + bacterial colonies (S. viridans)

Malden & Pai [7]		
*Patient 4*	Thickening of the left sinus floor and bone density alteration (suggestion of a retained root in the upper left second PM)	Sclerotic and necrotic bone

Khamaisi et al. [8]		
*Patient 5*	NA	NA

Marx et al. [9]		
*Patient 6*	NA	NA
*Patient 7*	NA	NA
*Patient 8*	NA	NA

Hamada [10]		
*Patient 9*	NA	NA

Barrow [11]		
*Patient 10*	Widening of the PLS	NA

Junquera et al. [12]		
*Patient 12a*	Generalized lytic pattern of bone destruction with superimposed sclerosis of the mandibular ramus	Necrotic osteitis + mixed infiltrate of lymphocytes and granulocytes + medular fibrosis + numerous Actinomyces colonies
*Patient 12b*		

Mehanna et al. [13]		
*Patient 13*	Marked right neck collection with free gas and midline shift	Normal skin flora with scanty diphtheroids

Favia et al. [14]		
*Patient 14a*	ID	NA
*Patient 14b*	ID	NA

Kwon et al. [15]		
*Patient 15*	Osteomyelitis characteristics	NA

Sedghizadeh et al. [16]		
*Patient 16*	Ill-defined lytic lesion	NA

Lo et al. [17]		
*Patient 17*	Sequestration osteosclerosis, focal osteolysis with cortical disruption	Actinomyces
*Patient 18*	Irregular area of bony sclerosis	NA

Shin et al. [18]		
*Patient 19*	Alveolar bone resorption with internal scattered residual bone fragments, widening of LPS, and radiolucent lesion	NA

Park et al. [19]		
*Patient 20*	Ill-defined lytic lesion	Necrotic bone + acute and chronic non- specific inflammation + granulation formation
*Patient 21*	Ill-defined lytic lesion	Necrotic bone
*Patient 22*	Ill-defined lytic lesion, mixed radiolucent and radiopaque lesions	NONE

Manfredi et al.[20]		
*Patient 23*		
*Patient 24*	NA	NA
*Patient 25*		
*Patient 26*		

Conte-Neto et al. [21]		
*Patient 27*	Radiolucent lesions and sequestration	Necrotic lamellar bone + chronic and acute inflammatory cells + bacterial colonies
*Patient 28*	Osteosclerosis and osteolysis	NA

*ID *Impossible to determine, *CLP *chronic lympho-plasmacytic, *NA *not available, *PLS *periodontal ligament space, *PM *premolar

**Table 4:** Management and Outcomes of BRONJ Lesions in Patients with RA

Authors	Management	Outcomes
Marunick et al. [4]		
*Patient 1*	ATB + Oral Rinses and sequestrectomy	No healing

Yarom et al. [6]		
*Patient 2*	ATB and curettage + debridement + resection	No healing
*Patient 3*	ATB and curettage + debridement + resection	Partial healing

Malden & Pai [7]		
*Patient 4*	ATB + CLX + DD and debridement	Progressive improvement

Khamaisi et al. [8]		
*Patient 5*	NA	NA

Marx et al. [9]		
*Patient6*	CLX + DD*	Complete healing
*Patient7*	CLX + DD*	Complete healing
*Patient 8*	CLX + DD*	Complete healing

Hamada [10]		
*Patient 9*	CT	Partial Improvement

Barrow [11]		
*Patient 10*	NA	NA

Junquera et al. [12]		
*Patient 12a*	ATB + CLX	Complete healing
*Patient 12b*	DD and sequestrectomy	Remission of symptoms

Mehanna et al. [13]		
*Patient 13*	ATB + DD + drainage	Complete healing

Favia et al. [14]		
*Patient 14a*	ATB + CLX + DD	Partial Improvement
*Patient 14b*	ATB + CLX + DD	Partial Improvement

Kwon et al. [15]		
*Patient 15*	Sequestrectomy and CT and resection and debridement + DD	Sequestrectomy: no healing. CT: complete healing in mx and no healing in Md. Resection and debridement: healing after 6 months

Sedghizadeh et al. [16]		
*Patient 16*	ATB + CLX + DD + sequestrectomy + debridement	NA

Lo et al. [17]		
*Patient 17*	ATB + CLX + DD and sequestrectomy + debridement	No healing
*Patient 18*	ATB + CLX + DD	No healing

Shin et al. [18]		
*Patient 19*	ATB + DD + CLX + debridement	Satisfactory healing

Park et al. [19]		
*Patient 20*	ATB + DD + CLX + curettage	Satisfactory healing
*Patient 21*	ATB + DD + CLX + curettage	NA
*Patient 22*	ATB + DD + CLX	Satisfactory healing

Manfredi et al. [20]		
*Patient 23*	DD	NA
*Patient 24*	DD + ATB + Laser	Partial healing
*Patient 25*	ATB + Laser	Partial healing
*Patient 26*	ATB	Partial healing

Conte-Neto et al. [21]		
*Patient 27*	ATB + DD + CLX + sequestrectomy + debridement	Complete healing
*Patient 28*	ATB + DD + CLX + curettage + debridement	Complete healing

*It was not possible to determine which ATB was prescribed to these patients

*ATB *antibiotic therapy, *CLX *chlorhexidine, *DD *drug discontinuation, *CT *conservative treatment, *NA *not available

### Patient characteristics

The patients from this literature review ranged in age from 55 to 79 yr and the mean age was 67 years. Among the 22 patients whose age was reported, 17 (77.3%) were above 60 years old [6,8,10-12,14-16,18-21], 25 patients reported the gender and only one was male [12] (Table 1). Furthermore, osteoporosis was the most common co-morbidity observed in this review, being present in 18 patients (66.6%) [4,9-11,14-16,18-21], followed by hypertension (29.6%) [6,16,18-20] and diabetes mellitus (14.8%) [8,15,20] (Table 1).

### Characteristics of bisphosphonate treatment

Among the 24 patients that reported the use of BPs, 22 (91.6%) affirmed to use alendronate [4,6-9,11,12,15,16,18-21], while 2 other patients were using ibandronate [14] or risedronate [10] (Table 1). The mean duration of BP therapy was 48 months, ranging from 6 months to 10 years, and most of the patients (73%) were using BPs for 3 or more years [4,6,9,12,15-17,19-21] (Table 1).

### BRONJ characteristics

Most of the oral BRONJ cases in this literature review were triggered by dental surgery procedures (51.7%), such as tooth extraction and dental implants, observed in 13 patients [4,5,7-9,12,14,17-20]. Of note, a large proportion of BRONJ lesions appeared spontaneously (41.38%) [9,10,12,13,15,19-21] (Table 2).

BRONJ lesions were located most commonly in the lower jaw (72.4%), especially in the posterior area [4,6,9,12-15,17,19,21]. The main signs and symptoms reported in the studies included: bone exposure in 82% of the lesions [4,6,7,9,11-18,20,21], followed by pain (78.5%) [4,6,7,10-14,16-21], edema (35.7%) [6,7,10,12,15,17-19,21] and purulent discharge (35.7%) [6,11,13-15,17,19,21]. Moreover, according to a staging system [9], 28 lesions were diagnosed at stage II or III, and only one was at stage I (Table 2). Furthermore, most of the patients (62.5%) presented a lytic pattern observed on image exams [4,12,16-19,21], followed by bone sclerosis that was reported in six patients (37.5%) [7,12,17,19,21].

Regarding the management of the BRONJ lesions, the studies showed the treatment either by conservative therapy alone (48%) [9,10,12,14,17,19,20] or associated with surgical procedures (52%) [4,6,7,12,13,15-19,21]. The treatment only with conservative therapy was associated with the most positive outcomes, including the complete healing of the lesions (33.3%) [9,12], partial and general positive results (58.3%) [10,14,19,20] and only one patient presented a non-healed lesion (8.33%) [17] (Table 4).

The association of conservative and surgical therapy showed more diverse results, with complete healing in 3 lesions (25%) [13,21], partial and general positive results (41.6%) [6,7,12,18,19] and 4 lesions were classified as non-healed (33.4%) [4,6,15,17] (Table 4).

## Discussion

Until the present moment, there is no randomized, controlled, prospective clinical trial that assessed jaw osteonecrosis (ONJ) risk in patients with rheumatoid arthritis who were using oral BPs. In the absence of this data, the present paper performed a critical literature review to investigate the current status of the epidemiological aspects of patients with rheumatoid arthritis affected by BRONJ.

Considering the prevalence of BRONJ lesions in patients with RA, we considered only the raw number of oral BRONJ reports in RA patients, since the accurate number of non-neoplasic patients treated with oral BPs is unknown; therefore, the determination of the precise prevalence of BRONJ is extremely difficult but is possible to be estimated based on epidemiological studies.

Two reviews published recently, reporting patients that presented BRONJ induced by the use of oral BPs, observed a very low prevalence of these lesions in patients with rheumatic diseases [22,23]. Other studies reported that the BRONJ prevalence in patients treated with oral BPs, ranges from 0.001% to 0.4% [17,24-27]. These numbers give the idea that the prevalence of BRONJ lesions in patients with RA is expected to be quite low, however it should be considered that the proportion of patients with rheumatic disease among all the population sampled in these studies is probably reduced as well.

At the same time, it is reasonable to expect an increased tendency in the number of BRONJ lesions in RA patients considering that there is a lack of knowledge about this disease among rheumatologists in many countries and that BPs are among the most frequent prescribed drugs in rheumatologic practice [28]. This is due to the high efficiency of BPs in the prevention and treatment of osteoporosis, which is a common feature in RA [29].

There is a considerable discussion in the literature whether aging plays a significant role in BRONJ development. Some studies found no statistically significant correlation between aging and BRONJ [30,31]. Therefore, the advanced age of the patients with BRONJ observed in the studies [6,8,10-12,14-16,18-21] may reflect nothing less than the increased BPs prescription to older patients compared with younger ones, since osteoporosis and RA are commonly seen in the elderly [29,32].

On the other hand, other authors include advanced age as a BRONJ co-factor [19,33,34], which could be related to the physiological effects of aging, including inflammatory issues [35], immune dysfunction [36], reduction of the blood flow and the remodeling ability [37,38], and increased oxidative stress [39]. In fact, these features are all implicated with BRONJ pathogenesis and could explain why this disease is not reported in young patients, even with other risk factors associated [40]. Paradoxically, BPs have been prescribed for the treatment of steroid-induced osteonecrosis of the joints in pediatric populations [41].

Controversial aspects have also been discussed regarding gender as a BRONJ co-factor. Some studies found no statistically significant correlation between gender and BRONJ [33,34]. Therefore, we observed that the large proportion of female patients from the studies [4,6-11,13-16,18-21] can represent only a coincidence, since women take oral BPs more frequently than males, especially because RA and osteoporosis are more common in women [42].

In spite of that, other authors reported a positive correlation between gender and BRONJ [19]. It has been speculated that estrogen therapy may play a role in this correlation, since hormonal reposition has been associated with an increased risk of BRONJ [43]. The concern is that hormonal replacement therapy is likely to occur in RA, since this disease is often worsened after estrogen delivery [44]. In fact, the association of BPs and estrogen reposition is especially possible in patients with no satisfactory outcomes associated with a single drug therapy or who have very low bone density with multiple risks [45]. Moreover, treatment with estrogen/bisphosphonate conjugate drugs has also been described [46].

It is not surprising that osteoporosis was the most prevalent co-morbidity observed in the studies [4,9-11,14-16,18-21], since this is a common feature in RA patients for several reasons, including: (a) post-menopausal women represent part of the main risk group for RA and are at risk for accentuated bone loss; (b) steroid therapy is often prescribed for the treatment of RA; (c) physical inactivity is characteristic of RA due to disease activity; and (d) bone loss due to inflammatory disease mechanisms, such as elevated levels of systemic cytokines [29].

It has been shown that there is a direct relation between BRONJ occurrence and BPs potency [13,33], which is supported by the high incidence of BRONJ in patients receiving intravenous BPs [47], as well as by faster lesion onset in these patients [24]. This assumption corroborates with the higher incidence of BRONJ in patients using alendronate, as seen in the studies revised here [4,6-9,11,12,15,16,18-21], since it is the most potent drug among the BPs [9].

In contrast, Kos and Luczak [48] found no correlation between the type of BPs and BRONJ incidence, which reinforces the assumption that it can be only a coincidence. In fact, over the last decade, alendronate was among the most used drugs in the USA as first-line therapy for the prevention or treatment of osteoporosis [49], both in non-neoplasic BRONJ patients without RA [6,9,14,16] and in patients with RA [32]. Furthermore, it is reasonable to understand why ibandronate, a potent nitrogen-containing bisphosphonate (N-BPs) [7] that is much less prescribed compared with alendronate, is not associated with large-series cases of BRONJ [23].

It has been discussed that one of the most critical factors for BRONJ is the duration of oral BPs therapy [9,33]. It is believed that there is an increased incidence of oral BRONJ in patients treated with oral BPs for more than 3 years [5,9], which is in agreement with the findings of the studies revised here [4,6,9,12,15-17,19-21]. In fact, longer-term use of oral BPs may have a dose-equivalence effect, potentially approximating BPs levels in bone thought to be achieved only through high-dose intravenous delivery [16], and the total dose administered over a long period of time is important for the magnitude of the bone turnover reduction [50].

An interesting observation is that it has been suggested a trend of early-onset lesions due to steroids therapy [9]. However, this tendency was not confirmed in the patients of the studies revised in this paper, since the mean duration of BP therapy in steroid-treated patients was similar to patients that have not been treated with this drug. Furthermore, some authors reported early-onset lesions in patients with RA and no history of steroids [3,19].

The literature indicates a strong association of BRONJ with dental surgical procedures in all groups of patients [3,5,9]. Although we also observed this tendency in the large number of lesions triggered by teeth extractions and dental implants in the studies that were retrieved [4,5,7-9,12,14,17-20], it is relevant to state that spontaneous occurrence of BRONJ lesions was observed in a significant proportion of individuals in several studies [9,10,12,13,15,19-21].

Most of the lesions in the studies were diagnosed in lower jaw at stage II or III [4,6-21] with the most common clinical findings being bone exposure, pain, edema, and purulent discharge. In fact, it also represents the same clinical aspects found in patients with no RA [6,9,14,16]. This evidence brings to light the lack of knowledge and attention about early clinical features that include non-specific signs and symptoms in the oral cavity, including no clinical evidence of bone exposure. The concern is that lesions can progress rapidly, and, in advanced stages, paresthesia, fistula formation, and pathologic fracture can also occur, even in RA patients [6].

The typical imaging finding in all groups of patients with BRONJ is the association of osteolysis and sclerosis aspects. Within this context, when it is considered that bone sclerosis is often detected in the initial stages of BRONJ [51], this sign can also be maintained in advanced stages; indeed, we observed in the studies that bone sclerosis was reported in six patients, all classified in advanced stages [7,12,17,19,21]. Therefore, a careful dental examination could allow an early diagnosis and intervention and assist the patients in responding more promptly to stage-specific treatment regimens for BRONJ.

Regarding the management and outcomes of BRONJ lesions in patients with RA, there is no widely accepted treatment protocol to BRONJ. Some authors believe that surgical procedures are not effective in patients with BRONJ and have led to further bone exposure, worsening of symptoms, and a greater risk of pathologic fracture, indicating conservative approaches as the best choice, including antibiotics, oral rinses with chlorhexidine, and 'drug holidays'[47]. In contrast, other authors believe that surgical procedures may achieve better outcomes in non-neoplasic patients [14].

In the papers that we revised, the lesions were treated either by conservative therapy (48%) or its association with surgical procedures (52%). Overall, this literature review showed that most of the lesions were treated with conservative approach [9,10,12,14,17,19,20] and were associated with the most positive outcomes including the complete healing of the lesions (33.3%) and no healing in only one patient (8.33%) [17] (Table 4). However, in other studies [4,6,7,12,13,15-19,21] the association of conservative and surgical therapy showed more diverse outcomes, with complete healing in only three lesions (25%) [13,21].

In fact, the management of BRONJ lesions is a great challenge, which reinforces the necessity of an adequate oral care through routine dental examinations, education and motivation of the patients to adopt preventive measures in order to maintain a good oral hygiene and [52].

## Conclusion

The main characteristics of BRONJ patients with RA are generally similar to those with no RA. This critical review highlights a serious concern regarding the delayed diagnosis of BRONJ lesions, since most of these patients were diagnosed in stage II/III. It is clear that the rheumatologist needs to be aware of the potential risk of their patients developing BRONJ and must work together with the dentist to prevent and detect the lesions as soon as possible.

## Competing interests

The authors declare that they have no competing interests.

## Authors' contributions

NCN analyzed the records, reviewed all patients' data. ASB drafted the manuscript and helped in writing the text. RACM and EMJ drafted the manuscript and reviewed it critically. All authors read and approved the final manuscript.

## Authors' information

NCN is a PhD student from Implantology program at Araraquara School of Dentistry and ASB is a PhD student from Periodontology program at Araraquara School of Dentistry. EMJ and RACM are professors and chairmen of the Department of Diagnosis and Surgery, Division of Periodontology at Araraquara School of Dentistry.
